# Synergistic anti-tumor efficacy of sorafenib and fluvastatin in hepatocellular carcinoma

**DOI:** 10.18632/oncotarget.15575

**Published:** 2017-02-21

**Authors:** Yang Cheng, RongCheng Luo, Hang Zheng, Biao Wang, YaHui Liu, DingLi Liu, JinZhang Chen, WanFu Xu, AiMin Li, Yun Zhu

**Affiliations:** ^1^ Digestive Department, Guangzhou Women and Children's Medical Center, Guangzhou Medical University, Guangzhou, Guangdong 510623, China; ^2^ Guangzhou Institute of Pediatrics, Guangzhou Women and Children's Medical Center, Guangzhou Medical University, Guangzhou, Guangdong 510623, China; ^3^ Cancer Center, Traditional Chinese Medicine-Integrated Hospital, Southern Medical University, Guangzhou, Guangdong 510315, China; ^4^ Department of Oncology, Nanfang Hospital, Southern Medical University, Guangzhou, Guangdong 510515, China; ^5^ Department of Hepatobiliary Surgery, Nanfang Hospital, Southern Medical University, Guangzhou, Guangdong 510515, China; ^6^ Liver Tumor Center, Nanfang Hospital, Southern Medical University, Guangzhou, Guangdong 510515, China

**Keywords:** sorafenib, fluvastatin, hepatocellular carcinoma, TLR4, SDF-1α

## Abstract

Drug resistance to sorafenib is common in patients with hepatocellular carcinoma(HCC). We examined the effects of a combination of sorafenib and fluvastatin on HCC using *in vitro* and *in vivo* models. The dual treatment induced apoptosis and reduced cellular viability in HCC more effectively than either drug alone. The combination treatment also inhibited activation of hepatic stellate cells, whereas single drug treatments did not. On a molecular level, combined treatment inhibited activation of the MAPK and NF-κB pathways via Toll-like receptor 4 in HCC cells. Combined treatment also inhibited expression of stromal cell-derived factor 1α in HCC cells, which further inhibited the MAPK pathway in hepatic stellate cells. These results suggest that a combination of sorafenib and fluvastatin may be a promising therapeutic strategy for patients with advanced HCC.

## INTRODUCTION

Hepatocellular carcinoma (HCC) is one of the most prevalent malignancies worldwide, and is the 5th and 7th most common cancer in men and women, respectively [1]. As HCC is characteristically refractory to current drug treatments, it is imperative to identify effective therapies for the treatment of advanced HCC.

Sorafenib was the first multikinase inhibitor to improve the overall survival of patients with advanced HCC. This drug inhibits tumor cell proliferation and angiogenesis and increases tumor cell apoptosis in HCC [2]. However, sorafenib only increases the survival time by a few months [3], and has a variety of adverse effects, including dermatological, digestive and cardiovascular toxicity [4]. These toxic effects may prompt the modification or discontinuation of sorafenib treatment. Thus, agents that can potentiate the anti-tumor activity of sorafenib without significant systemic toxicity need to be identified.

Fluvastatin, a member of the statin family, is widely used as a lipid-lowering drug. In addition to its cholesterol-lowering activities, fluvastatin has selective anti-cancer effects, such as the induction of tumor cell apoptosis in specific cancer cell lines (e.g., glioma and breast cancer cell lines) [5, 6]. Fluvastatin has also been suggested to have therapeutic potential for the treatment of HCC [7]. Notably, fluvastatin was shown to increase the cytotoxicity of sorafenib in melanoma cells [8]. However, the effects of a combination of sorafenib and fluvastatin in HCC have not been reported.

We hypothesized that co-administration of sorafenib and fluvastatin would have synergistic anti-tumor efficacy against HCC compared to treatment with either drug alone. In the present study, we investigated whether simultaneous treatment with low-dose sorafenib and fluvastatin would have synergistic anti-tumor effects in HCC cells and in an animal HCC model. The molecular pathways underlying these effects were also explored.

## RESULTS

### Synergistic anti-tumor effects of sorafenib and fluvastatin *in vivo*

**Figure 1:**
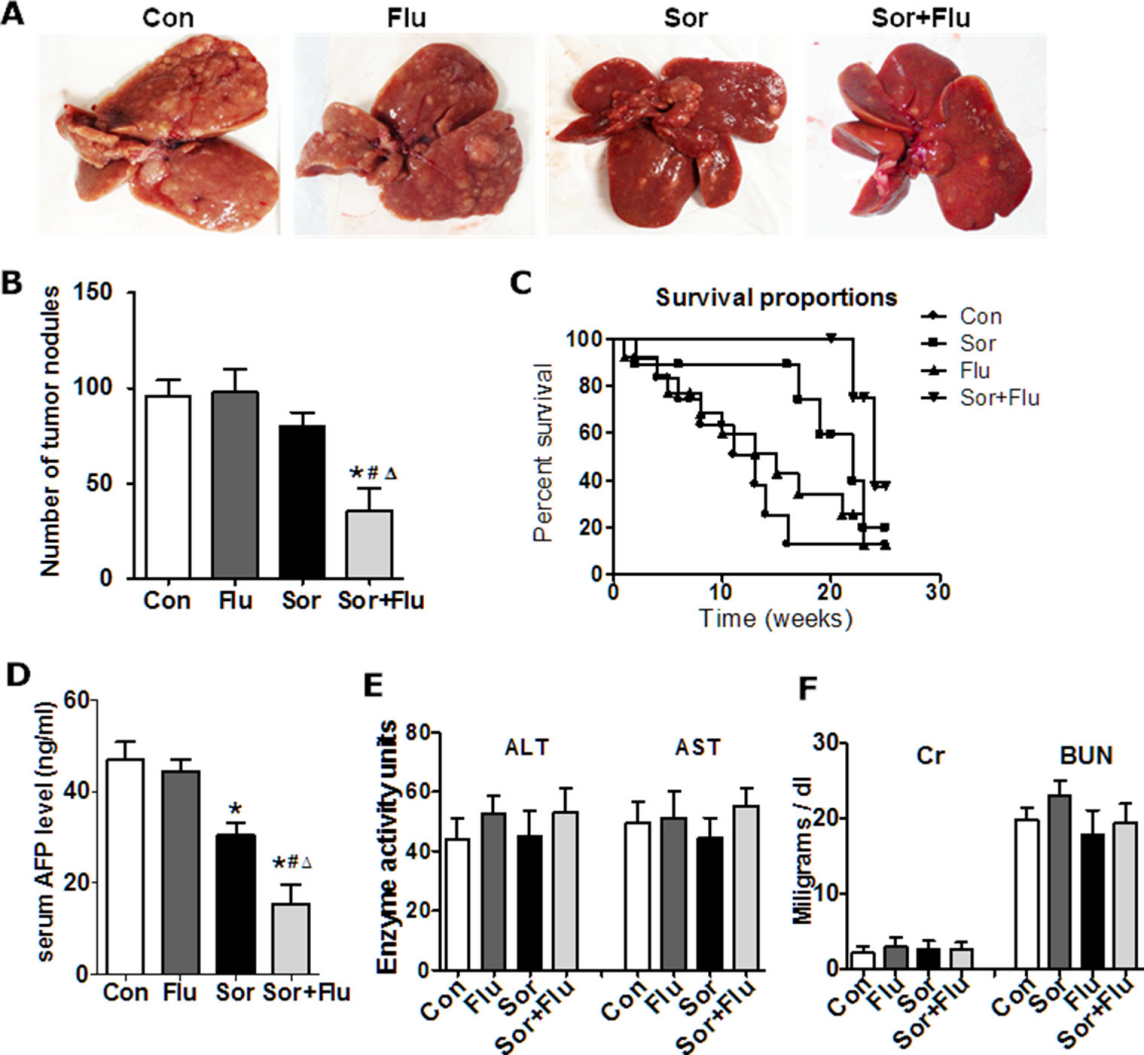
Sorafenib and fluvastatin reduced the progression of DEN-induced HCC (**A**) Representative photos of livers from the rat HCC model (*n* = 6). (**B**) Number of tumors larger than 3 mm in the rat livers. (**C**) Survival analysis for each treatment group (*n* = 12). (**D**) Serum AFP levels detected by ELISA. (**E**) Safety analysis was carried out. Liver toxicity was estimated based on AST and ALT levels. Kidney toxicity was evaluated by creatinine and blood urea nitrogen levels (**F**). The data are expressed as the mean ± SEM of three independent experiments. *P < 0.05 vs. Con.^#^*P* < 0.05 vs. Flu. ^Δ^*P* < 0.05 vs. Sor.

Low doses of sorafenib (10 mg/kg) and fluvastatin (10 mg/kg) were used in a diethylnitrosamine (DEN)-induced HCC rat model. The visible tumor nodules were reduced in the livers of rats of the combination group (*P* < 0.05, Figure 1A and 1B), but not of either of the single-treatment groups, compared with the control group (vehicle treated HCC rats). Survival analysis indicated that the survival time were longer in rats given the dual therapy than in those given the single-agent treatments (*P* < 0.05, Figure 1C). The serum alpha-fetoprotein (AFP) levels were also lower in the combination group than in the other groups (*P* < 0.05, Figure 1D). None of the rats displayed weight loss, bleeding in the skin or major organs, or significant hepatic or kidney injury (Figure 1E and 1F). These data suggested that the combination treatment more effectively inhibited tumor development in the rat HCC model than monotherapy.

### The dual treatment is superior in inhibiting tumor proliferation and increasing apoptosis

**Figure 2:**
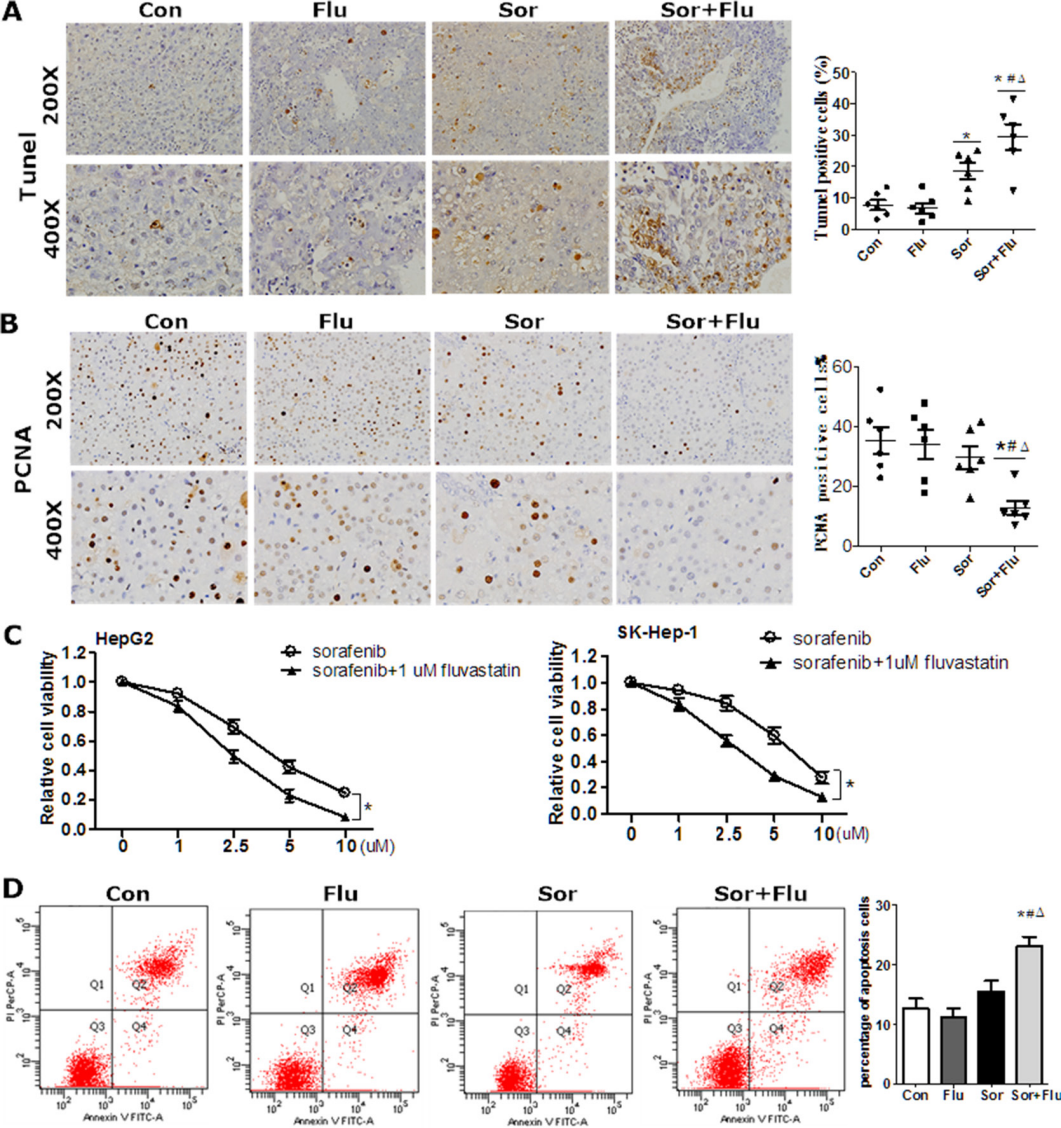
Synergistic cytotoxicity of sorafenib and fluvastatin against HCC (**A**) TUNEL assays of rat liver tissues. (**B**) IHC staining for PCNA in the rat liver (magnification, 200 × & 400 ×). (**C**) HepG2 and SK-Hep-1 cells were incubated various doses of sorafenib plus 1 μM fluvastatin or vehicle for 72 h. Cell viability was evaluated by CCK8 assays. *P < 0.05 vs. sorafenib group. (**D**) HepG2 and SK-Hep-1 cells were incubated with vehicle, 1μM sorafenib, 1 μM fluvastatin, or both agents for 48 h. Apoptosis was measured by Annexin V/PI. Annexin V-fluorescein isothiocyanate^+^ PI^−^ apoptotic cells are shown in the upper section. Data are expressed as the mean ± SEM of three independent experiments. *P < 0.05 vs. Con. ^#^*P* < 0.05 vs. Flu. ^Δ^*P* < 0.05 vs. Sor.

The effects of sorafenib and fluvastatin on the viability and apoptosis of HCC cells were evaluated both *in vivo* and *in vitro*. In the rat HCC model, a TUNEL assay revealed greater tumor necrosis in the combination group than in the other groups (*P* < 0.05) (Figure 2A). Cell proliferation was significantly lower in the combination group than in the control and monotherapy groups (*P* < 0.05, Figure 2B), as indicated by assays of proliferating cell nuclear antigen (PCNA)-positive cells. In HCC cell lines, sorafenib reduced cell viability in a dose-dependent manner (Figure 2C), but fluvastatin at 1 μM had no significant anti-tumor effects (Supplementary Figure 1). The combination of sorafenib and 1μM fluvastatin reduced cell proliferation to a greater extent than sorafenib alone (*P* < 0.05, Figure 2C). Low doses of sorafenib (1 μM) or fluvastatin (1 μM) had no effect on the apoptosis of HepG2 cells, as assessed by Annexin V/propidium iodide (PI), while the combination treatment significantly increased the level of apoptosis (Figure 2D). These results indicated that the dual therapy was more effective than monotherapy in inhibiting proliferation and inducing apoptosis in HCC.

### Effects of sorafenib plus fluvastatin on fibrosis and activation of hepatic stellate cells (HSCs) in HCC

**Figure 3:**
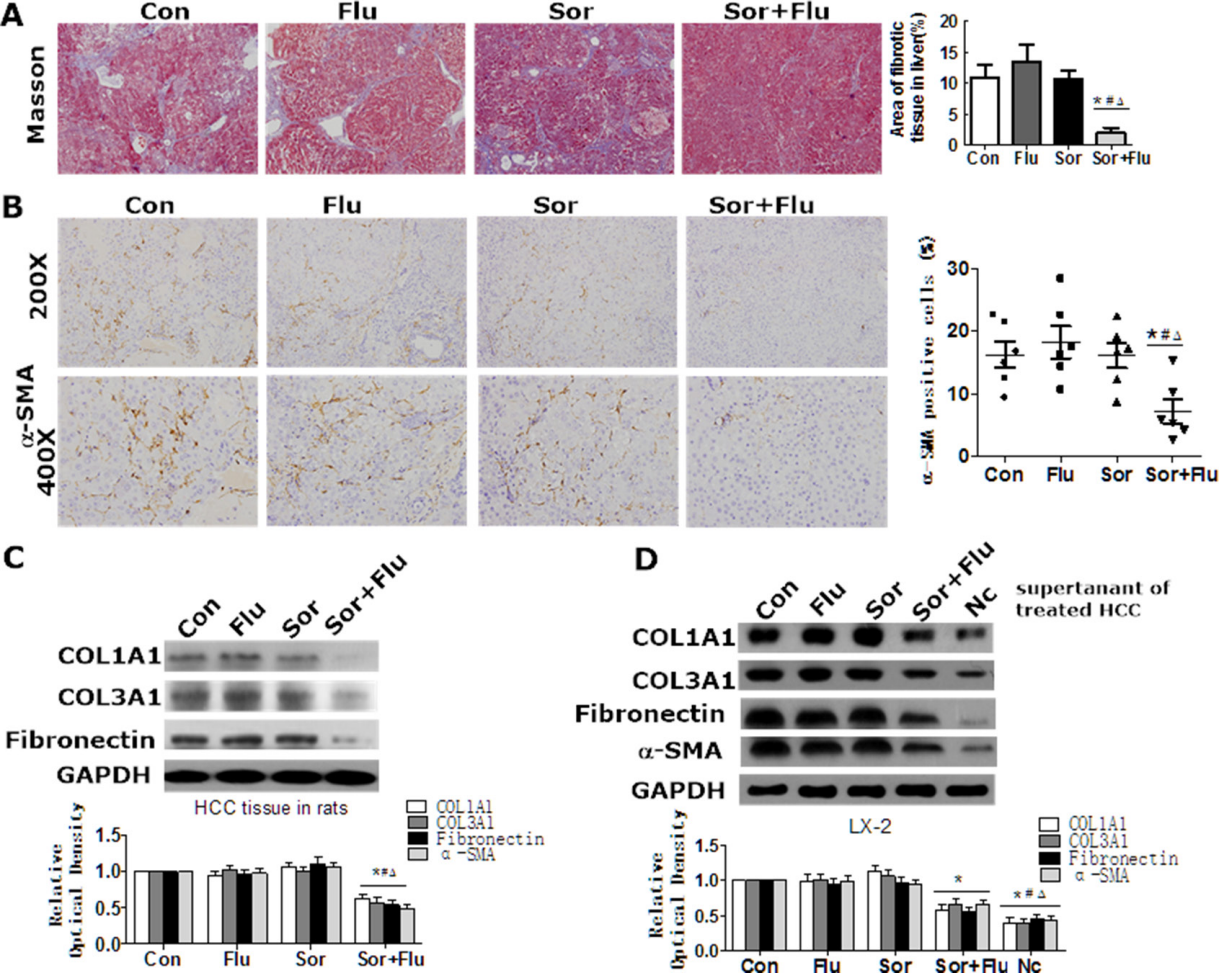
Effects of sorafenib and fluvastatin on liver fibrosis and HSCs activation (**A**) Representative Masson trichrome staining of rat liver sections. The fibrotic area was quantified with a computerized grid. (**B**) IHC of α-SMA in rat liver (magnification, 200 × & 400 ×). (**C**) The protein levels of COL1A1, COL3A1, and Fibronectin in the liver tissues of rats were detected by Western blotting. (**D**) HepG2 cells were treated with 1 μM sorafenib, 1 μM fluvastatin, 1 μM sorafenib plus 1 μM fluvastatin, or the vehicle. LX-2 cells were cultured in the supernatants of HepG2 cells or control medium (Nc) for 48 h. Western blotting was used to detect the levels of COL1A1, COL3A1, Fibronectin and α-SMA in LX-2 cells. Values are expressed as the mean ± SEM of at least three independent experiments. *P < 0.05 vs. Con. ^#^*P* < 0.05 vs. Flu. ^Δ^*P* < 0.05 vs. Sor.

Masson staining was performed to detect liver fibrosis in the rat HCC model. As shown in Figure 3A, DEN treatment induced notable accumulation of collagen in the livers of control rats compared with normal rats (*P* < 0.05). The accumulation of collagen in the liver was not reduced by monotreatment, but was significantly reduced by the co-treatment (Figure 3A). High α-smooth muscle actin (α-SMA) expression in the liver was observed in the control and monotherapy groups, while co-treatment significantly reduced α-SMA expression (Figure 3B). Similar results were found for the hepatic expression of COL1A1, COL3A1 and Fibronectin (Figure 3C).

We next assessed the *in vitro* effects of sorafenib and fluvastatin on the activation of HSCs. Low doses of sorafenib and fluvastatin had no effect on the proliferation, apoptosis or activation of HSCs (data not shown). However, the supernatants of control HCC cells significantly stimulated the activation of HSCs, while the supernatants of dual-treated HCC cells inhibited the activation of HSCs (Figure 3D). These results indicated that the combination treatment attenuated liver fibrosis and inhibited the activation of HSCs in HCC to a greater extent than monotherapy.

### Combination treatment significantly inhibits the expression of TLR4 in HCC

**Figure 4:**
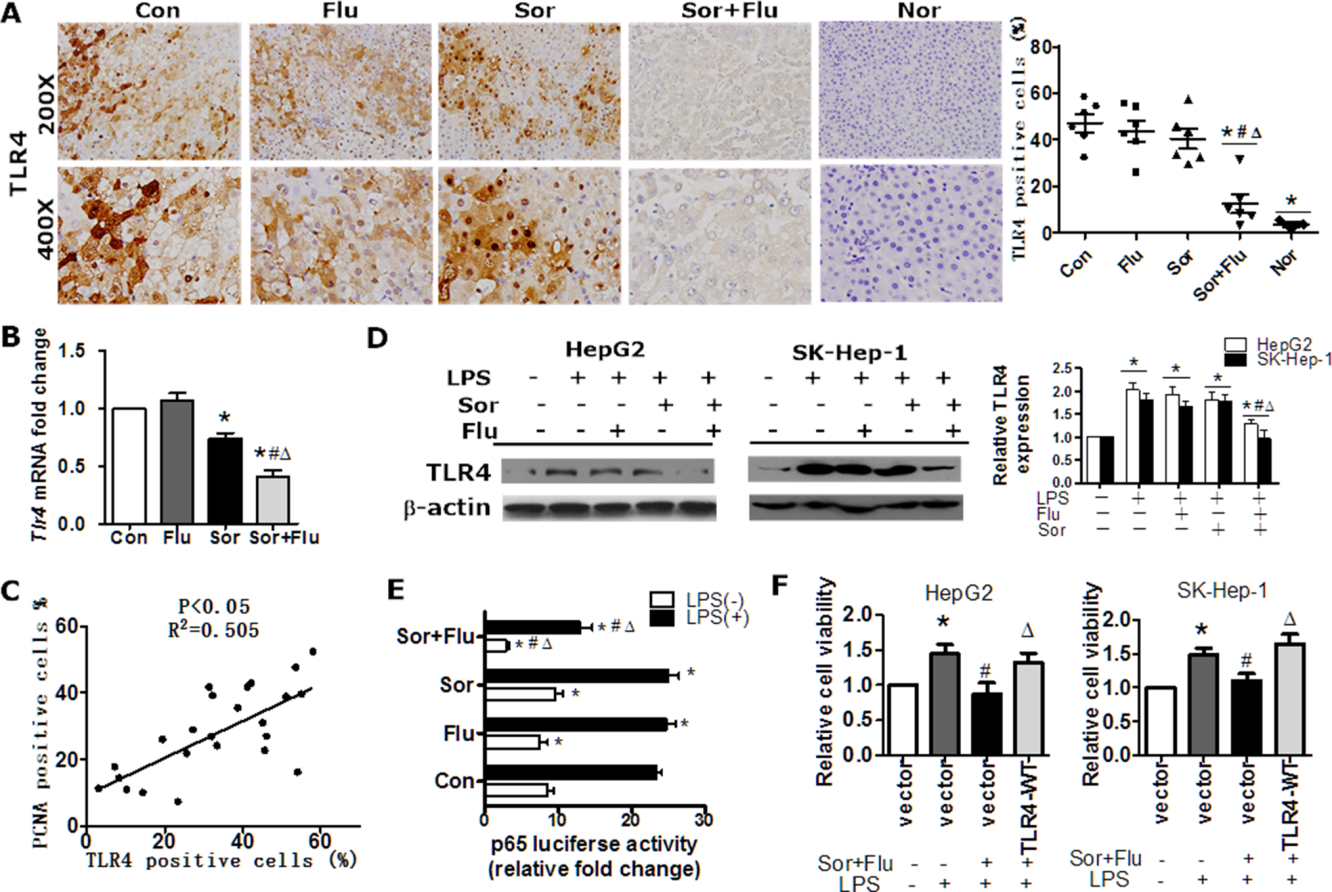
The combination treatment significantly inhibited TLR4 expression in HCC (**A**) IHC staining of TLR4 in rat liver tissues (magnification, 200 × & 400 ×). (**B**) The transcription of *Tlr4* in rat liver tissues was detected by qRT-PCR. *P < 0.05 vs. Con. ^#^*P* < 0.05 vs. Flu. ^Δ^*P* < 0.05 vs. Sor. (**C**) The correlation between TLR4 and PCNA expression in rat liver tumor tissues was determined by Pearson's χ^2^ test. (**D**) HepG2 and SK-Hep-1 cell lines were pre-treated with DMSO, sorafenib (1 μM) or fluvastatin (1 μM) for 2 h and then stimulated with 10 ug/mL LPS for 48 h. TLR4 expression was determined by Western blot analysis. *P < 0.05 vs. control. **P < 0.05 vs. LPS. ^#^*P* < 0.05 vs. LPS+Flu. ^Δ^*P* < 0.05 vs. LPS+Sor. (**E**) HepG2 cells were treated with 1 μM sorafenib, 1 μM fluvastatin, 1 μM sorafenib plus 1 μM fluvastatin, or the vehicle for 30 min, and then were incubated with vehicle or LPS (10 ug/mL) for 24 h. A luciferase assay was performed to detect p65 transcription factor binding activity. *P < 0.05 vs. Con. ^#^*P* < 0.05 vs. Flu. ^Δ^*P* < 0.05 vs. Sor. (**F**) HepG2 and SK-Hep-1 cells were transfected with the control vector or *TLR4*-WT, treated with DMSO or sorafenib (1 μM) plus fluvastatin (1 μM) 30 min prior to the addition of LPS (10 μg/mL), and cultured for another 72 h. Cell viability was determined by CCK8 assays. *P < 0.05 vs. control. ^#^*P* < 0.05 vs. LPS alone. ^Δ^*P* < 0.05 vs. LPS+Sor+Flu. Data are shown as the mean ± SEM of three independent experiments.

Toll-like receptor 4 (TLR4) contributes to HCC initiation and progression, as well as liver fibrosis [9]. TLR4 was overexpressed in human HCC tissues compared to normal liver tissues (*P* < 0.05, Supplementary Figure 2A). The mRNA and protein levels of TLR4 in HCC tissues were significantly lower in co-treated rats than in singly-treated rats (*P* < 0.05, Figure 4A and 4B). Moreover, TLR4 expression in HCC tissues correlated positively with PCNA-positivity in the rat model (Figure 4C). In HCC cell lines (HepG2 and SK-Hep-1 cells), lipopolysaccharide(LPS) stimulated the expression of TLR4 in a dose-dependent manner (Supplementary Figure 2B). TLR4 expression was significantly lower in HCC cell lines administered the combination treatment than in cells treated with a single agent (*P* < 0.05, Figure 4D). Dual treatment also reduced NF-κB p65 subunit binding to the *TLR4* promoter, as determined by a binding activity assay (Figure 4E). Further analysis revealed that ectopic TLR4 expression reversed the reduced cell viability following co-treatment (Figure 4F). These data suggested that sorafenib plus fluvastatin significantly inhibited expression of TLR4 in HCC.

### Sorafenib and fluvastatin coordinately inhibit the activation of the NF-κB and MAPK pathways by TLR4 in HCC

**Figure 5:**
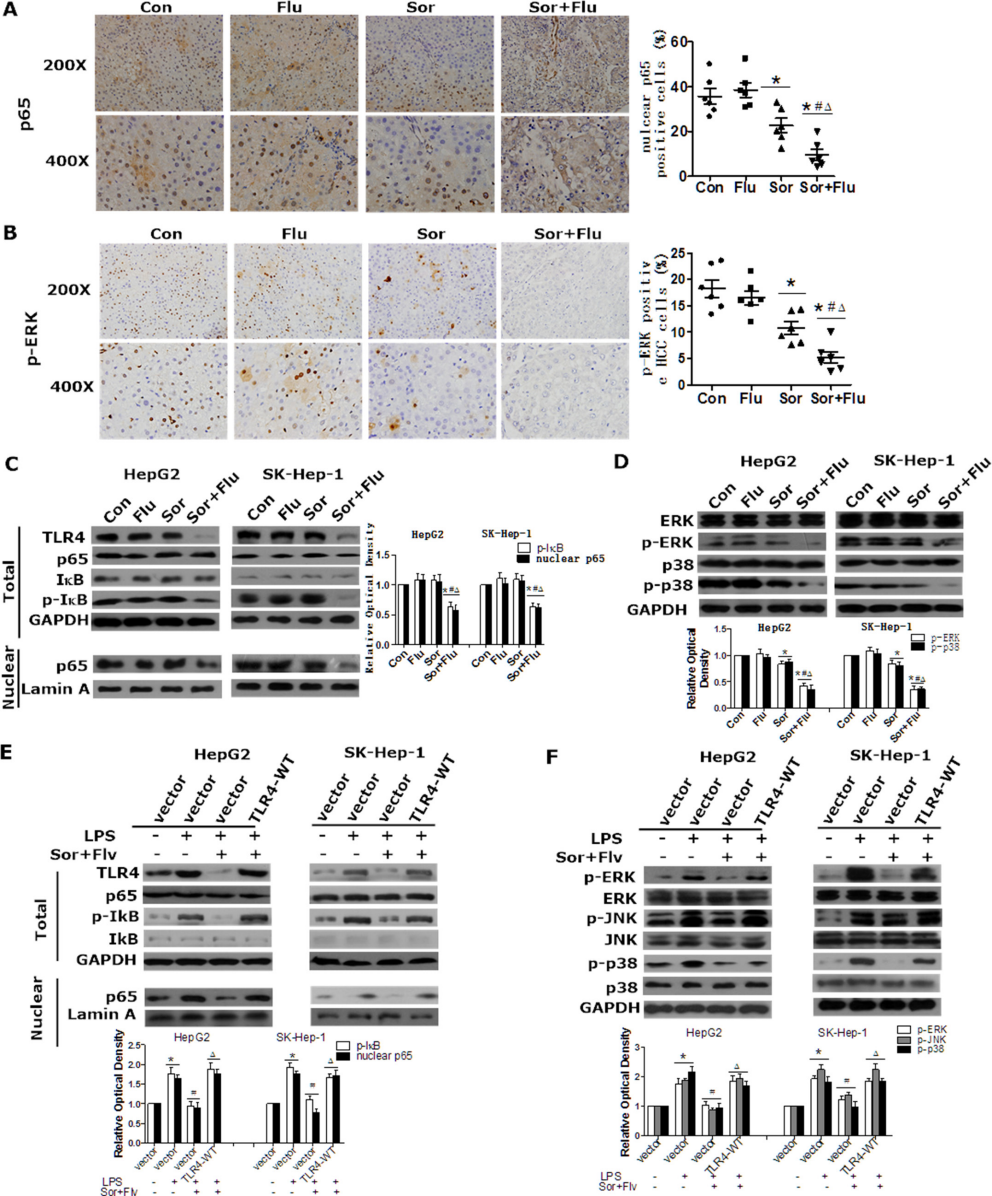
Sorafenib and fluvastatin inhibit the activation of the NF-κB and MAPK pathways in HCC The levels of p65 (**A**) and p-ERK (**B**) in rat liver tissues were detected by IHC (magnification, 200 × & 400 ×). (**C**)HepG2 and SK-Hep-1 cells were pretreated with sorafenib(1 μM), fluvastatin (1 μM) or both drugs for 24 h. Western blot analysis was performed to determine the protein level of p65. (**D**) Cells were treated as indicated in (C) for 30 min or 24 h. The levels of p-ERK, p-p38 (30 min), ERK and p38 (24 h) were examined by Western blot analyses. *P < 0.05 vs. Con. ^#^*P* < 0.05 vs. Flu. ^Δ^*P* < 0.05 vs. Sor. (**E**) HepG2 and SK-Hep-1 cells were transfected with the control vector or *TLR4*-WT. Twenty-four hours after transfection, cells were pre-treated with DMSO or sorafenib (1 μM) plus fluvastatin (1 μM) for 30 min and then induced by 10 ng/mL LPS for 24 h or 2 h. Western blotting was used to analyze the protein levels of p65, IκB (24 h) and p-IκB (2 h). The levels of p-ERK, p-JNK, p-p38 (2h), ERK, JNK and p38 (24 h) were also detected by Western blotting (**F**). *P < 0.05 vs. control. ^#^*P* < 0.05 vs. LPS alone. ^Δ^*P* < 0.05 vs. LPS+Sor+Flu. Data are shown as the mean ± SEM. The experiments were repeated three times.

NF-κB and MAPK are important proteins downstream of TLR4 that are critical stimulators of proliferation and inhibitors of apoptosis in HCC [10]. We further investigated whether sorafenib and fluvastatin would inhibit the NF-κB and MAPK pathways in HCC. In the rat HCC model, the phosphorylation of ERK and the nuclear translocation of p65 in liver tissues were significantly lower following co-treatment than following single drug administration (Figure 5A and 5B). In HCC cell lines, low doses of sorafenib or fluvastatin had no effect on p65, whereas co-treatment significantly suppressed the nuclear protein level of p65 (Figure 5C). The activity of the NF-κB p65 subunit in the nucleus exhibited similar results in an enzyme-linked immunosorbent assay (ELISA; Supplementary Figure 3A). The phosphorylation of ERK and p38 were also significantly inhibited in the co-treatment group (Figure 5D). Moreover, ectopic TLR4 expression reversed the inhibition of the MAPK and NF-κB pathways caused by co-treatment (Figure 5E, 5F and Supplementary Figure 3B). These data suggested that sorafenib and fluvastatin synergistically inhibited the activation of the NF-κB and MAPK pathways by TLR4 in HCC.

### Dual treatment inhibited the activation of HSCs by reducing stromal cell-derived factor (SDF)-1α expression in HCC

**Figure 6:**
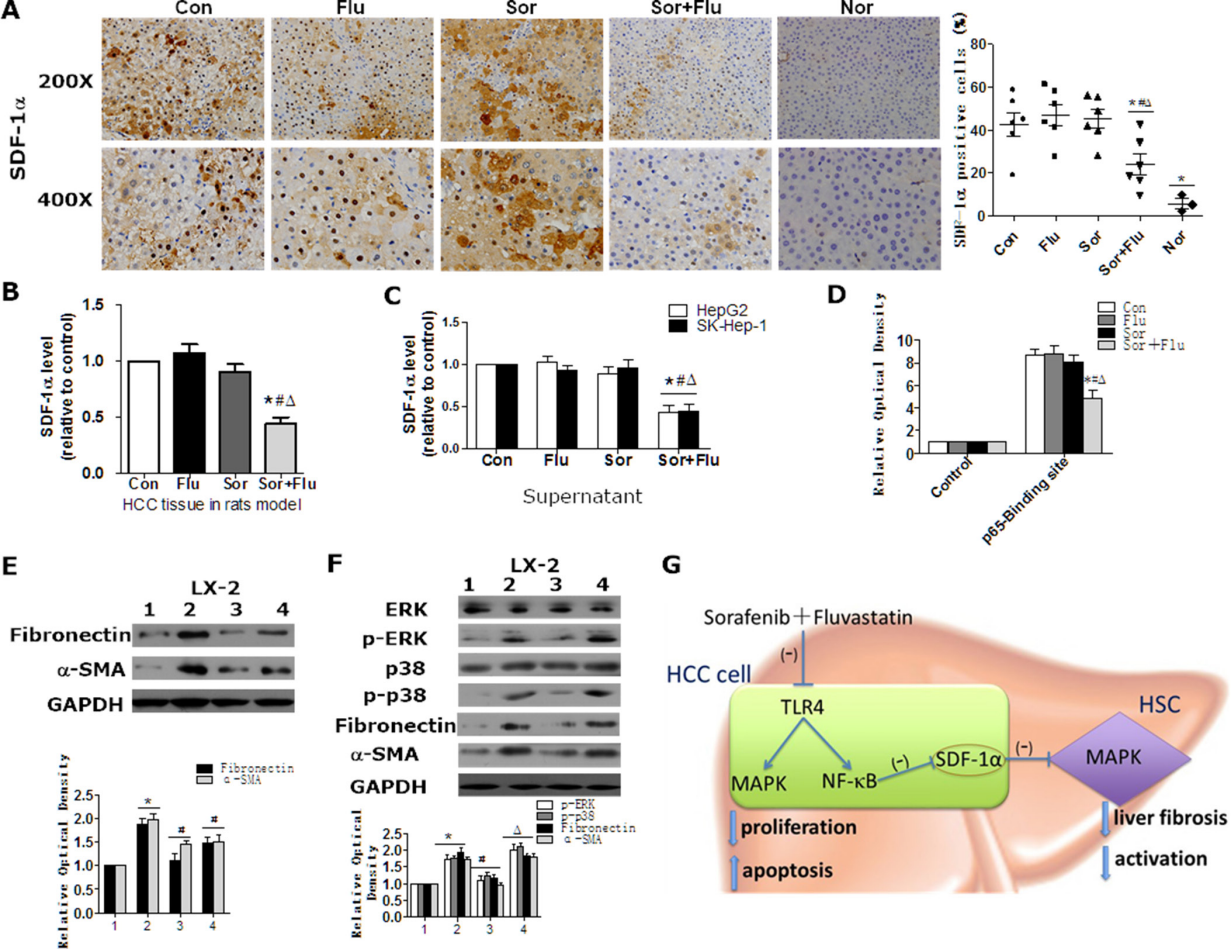
Sorafenib and fluvastatin inhibit the activation of HSCs by blocking the SDF1α/MAPK pathway SDF-1α expression in rat liver tissues was detected by IHC (**A**) and ELISA (**B**). (**C**) HepG2 and SK-Hep-1 cells were treated with sorafenib (1 μM), fluvastatin (1 μM) or both drugs for 24 h. ELISA was performed to determine the protein levels of SDF-1α in the culture supernatants. (**D**) HepG2 cells were treated as indicated in (C). The binding of p65 to *SDF-1α* was detected through ChIP assays. *P < 0.05 vs. Con. ^#^*P* < 0.05 vs. Flu. ^Δ^*P* < 0.05 vs. Sor. (**E**) LX-2 cells were treated with control medium (lane 1), supernatants from HepG2 cells transfected with control siRNA (lane 2), or supernatants from HepG2 cells transfected with *SDF-1α* siRNA-1 (lane 3) and *SDF-1α* siRNA-2 (lane 4) for 24 h. Western blot analysis was performed to detect the expression of Fibronectin and α-SMA. *P < 0.05 vs. lane 1. ^#^*P* < 0.05 vs. lane 2. (**F**) LX-2 cells were treated with control medium (lane 1), control supernatants (lane 2), or supernatants from dual-agent-treated (1 μM sorafenib+1 μM fluvastatin) HepG2 cells with (lane 3) or without rSDF1-α (40 ng/mL) (lane 4) for 30 min or 24 h. Western blot analysis was performed to detect the expression of p-ERK, p-p38 (30 min), ERK, p38, Fibronectin and α-SMA (24 h). *P < 0.05 vs. lane 1. ^#^*P* < 0.05 vs. lane 2. ^Δ^*P* < 0.05 vs. lane 3. Data are shown as the mean ± SEM of three independent experiments. (**G**) Schematic diagram describing the mechanism of combined sorafenib and fluvastatin in the treatment of HCC.

The SDF-1α/CXCR4 axis has been shown to directly stimulate HSCs differentiation to myofibroblasts in HCC by activating the MAPK pathway [11]. Inhibition of ERK with U0126 prevented the recombinant-SDF-1α (rSDF-1α)-induced upregulation of α-SMA and Fibronectin in HSCs, which confirmed that SDF-1α/MAPK can stimulate HSCs (Supplementary Figure 4A). We then investigated whether sorafenib and fluvastatin influenced SDF-1α levels. In the rat model, SDF-1α expression was increased in HCC tissues of the control group, while co-treatment significantly diminished the upregulation of SDF-1α (Figure 6A and 6B). In HCC cell lines, the secretion of SDF-1α was also significantly inhibited by co-treatment (Figure 6C). Chromatin immunoprecipitation (ChIP) assays demonstrated that fluvastatin and sorafenib synergistically inhibited the binding of NF-κB p65 subunit to the *SDF-1α* promoter (Figure 6D). These data suggested that sorafenib plus fluvastatin significantly inhibited SDF-1α expression in HCC cells.

The expression of SDF-1α correlated positively with α-SMA expression in rat HCC tissues (Supplementary Figure 4B). Culture supernatants from treated HCC cells were used to culture HSCs. The supernatants of HepG2 cells activated the MAPK pathway as well as the expression of Fibronectin and α-SMA in HSCs, whereas blocking SDF-1α with neutralizing antibody inhibited these effects (Supplementary Figure 4C). In addition, HSCs cultured with supernatants from specific *SDF-1α*-silenced HepG2 cells had significantly lower Fibronectin and α-SMA expression compared to those cultured with supernatants from control HepG2 cells (Figure 6E and Supplementary Figure 4D). Similar results were obtained in SK-Hep-1cells (data not shown). These data confirmed the involvement of SDF-1α in the activation of HSCs. The supernatants of dual-agent-treated HCC cells inhibited ERK phosphorylation and Fibronectin and α-SMA expression in HSCs, while rSDF-1α reversed these inhibitory effects (Figure 6F). Our data suggested that sorafenib plus fluvastatin inhibited the expression and secretion of SDF-1α, which subsequently blocked the MAPK pathway and suppressed the activation of HSCs.

## DISCUSSION

Drug resistance to sorafenib is common in patients with HCC. There is a growing need to explore combination treatments with sorafenib to enhance the anti-tumor effects on HCC. Fluvastatin has been suggested to have therapeutic potential for the treatment of HCC [7]. In the present study, we hypothesized that combining sorafenib and fluvastatin would maximize the therapeutic effects of these treatments. We used a low dose of sorafenib to assess its potential synergistic effects with fluvastatin [12]. We demonstrated that the combination treatment was superior to either drug treatment alone in promoting apoptosis and inhibiting proliferation in HCC, both *in vivo* and *in vitro*. In combination, these drugs also exhibited clear synergism in inhibiting liver fibrosis and activating HSCs. To our knowledge, this is the first report disclosing the synergistic effects of co-treatment with sorafenib and fluvastatin in HCC.

As an essential cell surface protein for LPS recognition, TLR4 can activate HSCs and promote liver fibrosis [13]. TLR4 activation is central to the progression of hepatic fibro-inflammatory disease and carcinoma [14–16]. High expression of TLR4 in HCC tissues has been shown to be strongly associated with early recurrence and poor survival in patients [17]. Consistent with a previous study [18], we found that TLR4 expression was greater in human HCC samples than in normal liver samples. The combination of sorafenib with fluvastatin significantly reduced *Tlr4* mRNA and protein levels in HCC cells, both *in vitro* and *in vivo*. These synergistic effects may have been due to the inhibition of p65 binding to the *TLR4* promoter.

The NF-κB and MAPK pathways are major stimulators of proliferation and inhibitors of apoptosis in HCC. Consistent with previous studies [15, 19, 20], we found that LPS/TLR4 signaling activated the NF-κB/MAPK pathways. Moreover, co-treatment synergistically inhibited the LPS/TLR4-activated NF-κB/MAPK pathways in HCC cells.

The interaction between HCC cells and HSCs is central to tumor progression. HCC cells secrete cytokines and chemokines to promote the activation of HSCs, while activated HSCs also produce chemokines to promote tumor cell proliferation and migration [21]. A recent study demonstrated that HSCs induce HCC resistance to sorafenib [22]. This indicates the potential involvement of tumor fibrosis in the development of resistance to sorafenib in HCC. Sorafenib has dual effects on HSCs and liver fibrosis in HCC progression. Despite its inhibitory effect on hepatic fibrogenesis, sorafenib may activate the SDF-1/CXCR4 pathway and increase tumor-associated fibrosis in HCC [11, 23]. Fluvastatin has been shown to attenuate hepatic steatosis-induced liver fibrosis by inhibiting the paracrine effects of hepatocytes on HSCs [24]. However, the effect of fluvastatin on liver fibrosis in HCC has never been determined. In our study, low doses of sorafenib or fluvastatin alone could not attenuate fibrogenesis in HCC, while dual treatment significantly alleviated fibrosis by inhibiting the activation of HSCs both *in vivo* and *in vitro*.

SDF-1α is a potent chemoattractant for fibrocytes and thus contributes to liver fibrosis. The SDF-1α/CXCR4 pathway directly induces HSC differentiation and proliferation by activating MAPK to increase tumor-associated fibrosis [11]. Consistent with recently reported findings, we found that SDF-1α was overexpressed in HCC and could activate HSCs by stimulating the MAPK pathway. Sorafenib has been shown to elevate SDF-1α/CXCR4 in a mouse HCC model [23]. However, in our DEN-induced rat HCC model, sorafenib treatment did not alter the expression of SDF-1α in HCC cells. This difference may have been due to the low dose of sorafenib used in our study. Single treatment with fluvastatin also had no effect on SDF-1α expression in HCC. However, dual treatment significantly inhibited the expression of SDF-1α by preventing the binding of p65 to the *SDF-1α* promoter in HCC cells. Our data indicated that sorafenib plus fluvastatin inhibited the expression and secretion of SDF-1α in HCC cells and subsequently reduced the activation of HSCs by inhibiting the MAPK pathway.

In conclusion, the combination of sorafenib and fluvastatin significantly inhibited cellular proliferation and promoted apoptosis in HCC by inhibiting the TLR4-activated NF-κB and MAPK pathways. Dual treatment also significantly alleviated tumor-associated fibrosis and inhibited the activation of HSCs by blocking the expression and secretion of SDF-1α in HCC cells, and subsequently inhibited the MAPK pathway in HSCs (Figure 6G). Our results indicate that the combination of sorafenib and fluvastatin may be a potential therapeutic strategy for patients with advanced HCC.

## MATERIALS AND METHODS

### Chemicals

For *in vitro* experiments, sorafenib (Selleck Chemicals, Houston, TX, USA) and fluvastatin (Sigma, St. Louis, MO, USA) were dissolved in DMSO. For *in vivo* experiments, sorafenib was dissolved in a Cremophor EL-ethanol solution (50:50, Sigma Cremophor EL :95% ethyl alcohol) and diluted with distilled water [7], while fluvastatin was dissolved in distilled water.

### Animal studies

All protocols for animal care and use were approved by the Animal Care Committee of the Southern Medical University. Male Wistar rats received intraperitoneal injections of DEN (Sigma) at 50 mg/kg weekly for 12 weeks. At the end of three months of DEN injection, three rats were randomly chosen and killed to ensure the development of hepatocellular carcinoma, as confirmed by pathological analysis. Rats were then submitted to daily gavage administration of either 10 mg/kg sorafenib (Group Sor), 10 mg/kg fluvastatin (Group Flu), a combination of both substances (Group Sor+Flu), or vehicle (Group Con). Vehicle-injected normal rats were used as controls (Group Nor). After 8 weeks of drug treatment, rats were sacrificed for analysis (*n* = 6) or continuously treated as indicated for survival analysis(*n* = 12). Malignant liver nodules ≥ 3 mm in diameter were counted by two independent investigators. Liver tissue and serum samples were collected for later analysis.

### Human tissue samples

The use of human samples was approved by the patients and the ethics committee of Nanfang Hospital, Southern Medical University. Fifty liver tumor tissue samples from patients with HCC at Nanfang Hospital were confirmed by pathology. Thirty normal liver tissue samples were also collected. The patients’ characteristics are shown in Table 1.

**Table 1:** Characteristics of patients with HCC

Characteristic		No. of patients	Percentage
Gender	Male	32	64
	Female	18	36
Age	≤ 45	23	46
	>45	27	54
Pathology	High differentiated	15	30
	Moderately differentiated	24	48
	Low differentiated	11	22
AFP	≤ 400	18	36
	> 400	32	64
Tumor size	≤ 5 cm	25	50
	> 5 cm	25	50
Prognosis	Recrudesce	35	70
	No-recrudesce	15	30

### Immunohistochemistry (IHC)

IHC staining was performed with standard protocols [25]. Specific primary antibodies against TLR4, phospho-ERK (p-ERK), p65, α-SMA (Abcam, Cambridge, UK), and SDF-1α (Novus Biologicals, Littleton, CO, USA) were used. The results are expressed as the percentage of positively stained cells in five randomly selected microscopic fields counted by two independent researchers.

### Cell culture

The human hepatic stellate cell line LX-2 and the human HCC cell lines HepG2 and SK-Hep-1 were obtained from Shanghai Advanced Research Institute, Chinese Academy of Sciences. Cells were maintained in DMEM supplemented with 10% fetal bovine serum and incubated in humidified air containing 5% CO_2_ at 37°C. Cultures were transfected with Lipofectamine 2000 (Invitrogen, Carlsbad, CA, USA) according to the manufacturer's instructions for the following experiments. To obtain conditioned media for the culture of LX-2 cells for specific experiments, we treated HepG2 and SK-Hep-1 cells with sorafenib/fluvastatin for 24 h and washed them three times with PBS. After routine culture of the cells for 48 h, the supernatants were collected.

### Cell viability assay

HepG2 and SK-Hep-1 cells were seeded in 96-well plates (2000 cells/well) and were treated with fluvastatin or sorafenib for 72 h. The relative numbers of viable cells were detected by CCK8 reagents (DingGuo Bio, Beijing, China) [26].

### TUNEL assay

Apoptotic cells in HCC tissues from rats were analyzed with a Click-iT^™^ TUNEL Colorimetric IHC Detection Kit (Thermo Fisher, Hudson, NH, USA) in accordance with the manufacturer's instructions. TUNEL-stained apoptotic cells were counted in five randomly selected microscopic fields.

### Apoptosis assay

Pretreated HCC cells were stained with an Annexin V-fluorescein isothiocyanate/PI apoptosis detection kit (BD Biosciences, La Jolla, CA, USA) according to the manufacturer's instructions. Stained cells were analyzed by FACSCalibur.

### Gene expression analysis

Total RNA was extracted and cDNA was synthesized according to standard protocols. The cDNA was further PCR-amplified by specific primers with an All-in-One qPCR Mix kit (GeneCopoeia, Rockville, MD, USA). *Gapdh* was used as a housekeeping gene. The primers used are listed in Supplementary Table 1.

### Western blotting

Protein extracts were obtained through standard protocols [27]. Western blot analyses were performed as described previously [27]. Specific primary antibodies against COL1A1, COL3A1, Fibronectin, α-SMA, ERK, IκB, phospho-IκB, JNK, phospho-JNK (Cell Signaling Technology, Danvers, MA, USA), TLR4, p-ERK, p65 and SDF-1α (Abcam) were used. β-actin (Abcam) and Lamin A (Santa Cruz) were used as loading controls for total protein and nuclear protein, respectively.

### ELISA

Rat serum AFP levels were determined by ELISA (Bio-Swamp, Shanghai, China) per the manufacturer's instructions. The NF-κB nuclear concentrations and SDF-1α levels in the culture supernatants of treated HCC cells were also detected by ELISA (Abcam).

### Luciferase reporter assay

Lipofectamine 2000 was used to transfect HepG2 cells with a pGL3 [luc/SV40] reporter plasmid containing the promoter sequence of hTLR4, or a pGL4.74 [hRluc/TK] control plasmid. Cells were subsequently incubated with different agents for 24 h. The firefly and *Renilla* luciferase activities of the cell extracts were measured with a Dual-Luciferase Reporter Assay System (Promega, Madison, WY, USA) according to the manufacturer's instructions.

### ChIP

P65 binding to the *SDF-1α* promoter was tested by ChIP analysis according to the manufacturer's instructions. Promoter binding was examined by RT-PCR with the following primers: *SDF-1α*, F: TCTAGGGAAGTTCCAAACAGGAG; R: ACCAG GGAGCCATATTTTGGG.

### Statistical analysis

All experimental data are expressed as the mean ± SEM from at least three experiments. Unpaired Student's *t*-tests and analysis of variance (ANOVA) were used for statistical analysis. All statistical analyses were conducted with SPSS 13.0 software. A *P*-value less than 0.05 was considered statistically significant.

## SUPPLEMENTARY MATERIALS FIGURES AND TABLES


