# Supplementary material for: Synergistic anti-tumor efficacy of sorafenib and fluvastatin in hepatocellular carcinoma
Source: Oncotarget. 2017 Feb 21;8(14):23265–76. doi: 10.18632/oncotarget.15575 (PMC5410302; doi:10.18632/oncotarget.15575)
Supplement: Supplementary file 1 [file oncotarget-08-23265-s001.pdf]

Synergistic anti-tumor efficacy of sorafenib and fluvastatin in hepatocellular carcinoma

Supplementary Materials

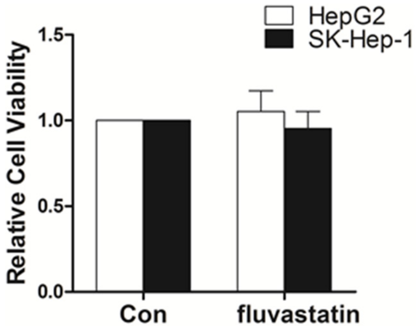

Supplementary Figure 1: HepG2 and SK-Hep-1 cells were incubated with 1µM fluvastatin or vehicle for 72 h. Cell viability was evaluated by CCK8 assays. *P < 0.05 vs. control group. Data are expressed as the mean ± SEM of three independent experiments.

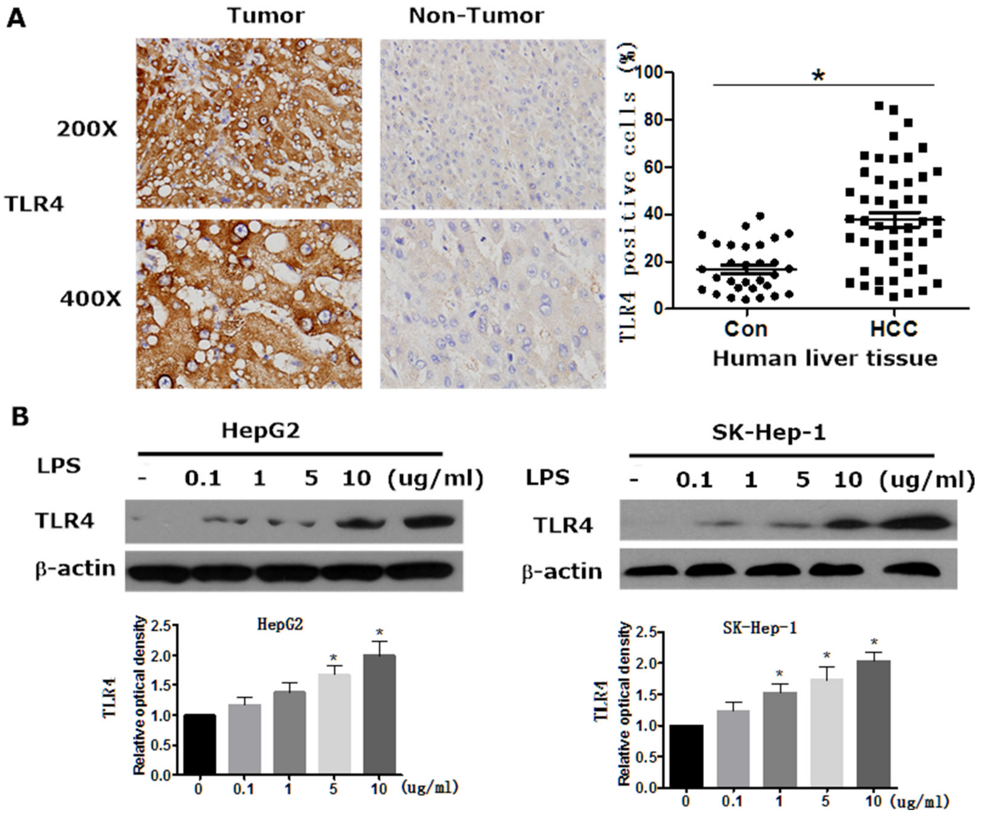

Supplementary Figure 2: (**A**) IHC staining of TLR4 in human HCC tissues and normal liver tissues. *P < 0.05 vs. non-tumor samples. (**B**) The serum-starved HepG2 and SK-Hep-1 cell lines were treated with LPS (0, 0.1, 1, or 10 µg/ml) for 24 h. The protein level of TLR4 was determined by western blot analysis. Data are expressed as the mean ± SEM of three independent experiments. *P < 0.05 vs control.

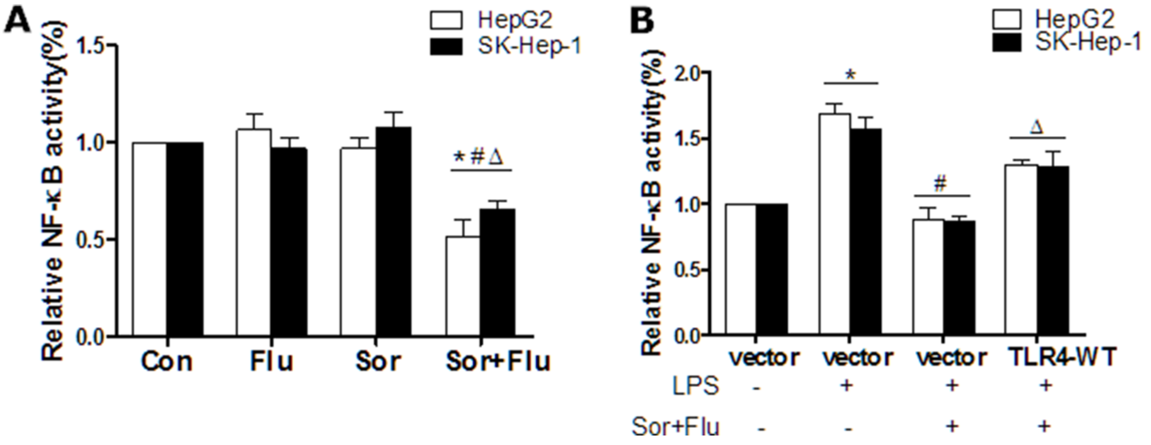

Supplementary Figure 3: (**A**) HepG2 and SK-Hep-1 cells was pretreated with sorafenib (1 μM), fluvastatin (1 μM) or both drugs for 24 h. The protein level of p65 in the nucleus was determined by ELISA. **P* < 0.05 vs. Con, #*P* < 0.05 vs. Flu and Δ*P* < 0.05 vs. Sor. (**B**) HepG2 and SK-Hep-1 cells were transfected with control vector or TLR4-WT. Twenty-four hours after transfection, HCC cells were pre-treated with DMSO or sorafenib (1 μM) plus fluvastatin (1 μM) for 30 min and then induced with 10 ug/mL LPS for 24 h. Changes in p65 activity were determined by ELISA. Data are expressed as the mean ± SEM of three independent experiments. **P* < 0.05 vs. control, #*P* < 0.05 vs. LPS alone and Δ*P* < 0.05 vs. LPS+Sor+Flu.

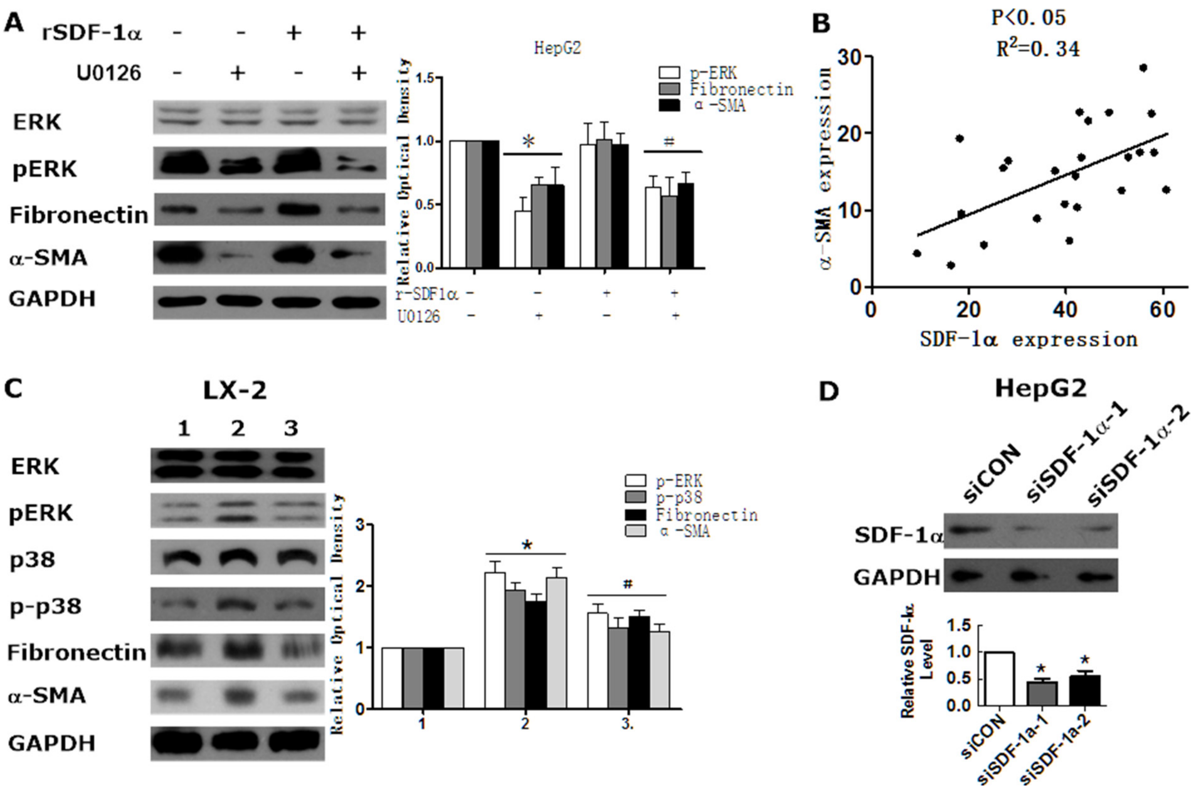

Supplementary Figure 4: (**A**) LX-2 cells were cultured with U0126 (2 μM) or rSDF1-α (40 ng/ml) as indicated for 30 min and 24 h. The expressions of p-ERK, p-p38, fibronectin and α-SMA were measured by western blotting. **P* < 0.05 vs. control, #*P* < 0.05 vs. rSDF1-α treated group. (**B**) The correlation between SDF-1α and PCNA expression in liver tumor tissues of HCC rats was determined by Pearson's χ^2 test. (**C**) LX-2 cells were treated with control medium (lane 1), culture supernatant of HepG2 cells (lane 2), or supernatant of HepG2 cells plus neutralizing antibody of SDF-1α (lane 3) for 30 min or 24 h. Western blot analysis was performed to detect the expression of p-ERK, p-p38 (30 min), ERK, p38, fibronectin and α-SMA (24 h). **P* < 0.05 compared to lane 1, #*P* < 0.05 vs. lane 2. (**D**) HepG2 cells were transfected with control siRNA, SDF-1α siRNA-1 and SDF-1α siRNA-2. The protein level of SDF-1α was detected by Western blotting. **P* < 0.05 vs. siCON. Data are expressed as the mean ± SEM of three independent experiments.

Supplementary Table 1: Sequence of primers for qRT-PCR

Gene	Forward primer	Reverse primer
Tlr4	5′- TGTATCGGTGGTCAGTGTGC -3′	5′-CAGCTCGTTTCTCACCCAGT -3′
Gapdh	5′-TGTTCGTCATGGGTGTGAAC-3′	5′-ATGGCATGGACTGTGGTCAT-3′
